# MicroRNA-682-mediated downregulation of PTEN in intestinal epithelial cells ameliorates intestinal ischemia–reperfusion injury

**DOI:** 10.1038/cddis.2016.84

**Published:** 2016-04-28

**Authors:** Z Liu, J Jiang, Q Yang, Y Xiong, D Zou, C Yang, J Xu, H Zhan

**Affiliations:** 1 Division of Emergence Medicine, Department of General Internal Medicine, The First Affiliated Hospital of Sun Yat-Sen University, No. 58, Zhongshan 2nd Road, Guangzhou 510080, China; 2 Department of Gastroenterology, The Third Affiliated Hospital of Sun Yat-Sen University, No. 600, Tianhe Road, Guangzhou 510360, China

## Abstract

Intestinal ischemia–reperfusion (I/R) injury causes inflammation and tissue damage and contributes to high morbidity and mortality, but the underlying mechanism remains elusive and effective therapies are still lacking. We report here a critical role of the microRNA 682 (miR-682) as a key regulator and therapeutic target in intestinal I/R injury. MiR-682 was markedly induced in intestinal epithelial cells (IECs) during intestinal ischemia in mice and in the human colonic epithelial cells during hypoxia, but was undetected rapidly after intestinal reperfusion in IEC of mice. MiR-682 induction during hypoxia was modulated by hypoxia-inducible factor-1*α* (HIF-1*α*). On lentivirus-mediated miR-682 overexpression *in vivo* during intestinal reperfusion or miR-682 mimic transfection *in vitro* during hypoxia, miR-682 decreased the expression of phosphatase and tensin homolog (PTEN) and subsequently activated nuclear translocation of nuclear factor kappa B (NF-*κ*B) p65. Consequently, NF-*κ*B activation by miR-682-mediated PTEN downregulation prevented reactive oxygen species (ROS) induction, inflammatory reaction, mitochondrial-mediated apoptosis and IEC apoptosis. The effect of miR-682-mediated PTEN/NF-*κ*B pathway on IECs resulted in protection against intestinal I/R injury in mice. However, NF-*κ*B chemical inhibitor reversed miR-682-mediated decreased PTEN expression, ROS induction, inflammation and IEC apoptosis. Collectively, these results identify a novel miR-682/PTEN/NF-*κ*Bp65 signaling pathway in IEC injury induced by I/R that could be targeted for therapy.

MicroRNAs (miRNAs) are endogenously produced short and noncoding RNAs which are important regulator of target messenger RNA translation by binding mainly to complementary sequences of the 3′ untranslated region of mRNA transcript, thereby leading to RNA degradation and/or inhibition of protein synthesis.^1, 2, 3^ miRNAs have been found to be not only crucial for development and the maintenance of physiological homeostasis, but have also been causally implicated in diverse pathological condition.^4, 5^ Numerous researches have proved that miRNAs play an indispensable role in gastrointestinal development and physiology.^6, 7, 8^ Moreover, characteristic changes in miRNA expression are associated with cell apoptosis, oxidative stress and inflammation.^7, 9, 10^ For example, IEC damage induced by immunological dysfunction are closely associated with marked expression alterations in miR-21, miR-422b and miR-23a, which contribute to IEC apoptosis, abnormal permeability and intestinal fibrosis by targeting specific downstream genes.^11^

Small intestinal epithelium normally renews every 5 days with highly differentiated cells at the tip of villi removed by apoptosis, a physiological process for eliminating unwanted or damaged cells.^12, 13^ The small intestines with well-featured renewal process have been believed as an excellent model to research how tissue homeostasis function through the balance of cell birth and cell death.^14, 15, 16^ Apoptosis has also been demonstrated to have an important impact in tissue injury under some pathological conditions, such as ischemia–reperfusion (I/R) induced injury in intestines, brain, myocardium and liver.^17, 18^ Many evidence reveals that apoptosis is a major mode of cell death caused by I/R in these tissues. The small intestine is prone to be subjected to ischemic-induced apoptosis because of the priority of blood flow supplied to brain and heart when hypovolemic shock occurs. Several cell signaling pathways have been found to be involved in intestinal I/R-induced apoptosis. For example, the increase of Toll-like receptors signaling molecular interleukin 1 receptor-associated kinase (Irak) 1 promoted by miR-146a fortified epithelial ligand responsiveness, chemokine secretion, apoptosis and mucosal barrier disruption in experimental intestinal I/R model.^19^ Inhibition of Notch signaling by chemical inhibitor or siRNA could enhance the apoptosis of IEC-6 cells under the condition of hypoxia.^20^ Pharmacological blockade of protein kinase C *β*_2_ induced by intestinal I/R markedly protected small intestine against oxidative-stress-stimulated apoptosis.^21^ PI3K/AKT pathway was activated to protect against intestinal I/R injury.^22^ However, the underlying molecular mechanism of I/R-induced intestinal apoptosis has remained poorly understood.

In the present study, we found changes of 9 miRNA expression of 30 novel miRNAs chosen from the expression profile of miRNAs in mouse embryos^23^ in intestinal I/R injury. Moreover, we have identified miR-682 as a key miRNA that is markedly induced during intestinal I/R injury and targets phosphatase and tensin homolog (PTEN) to activate nuclear factor kappa B (NF-*κ*B) p65 pathway, which in turn, reduces IEC apoptosis and prevents intestinal I/R injury.

## Results

### Upregulation of miR-682 in IEC of mice during intestinal ischemia

**Figure 1:**
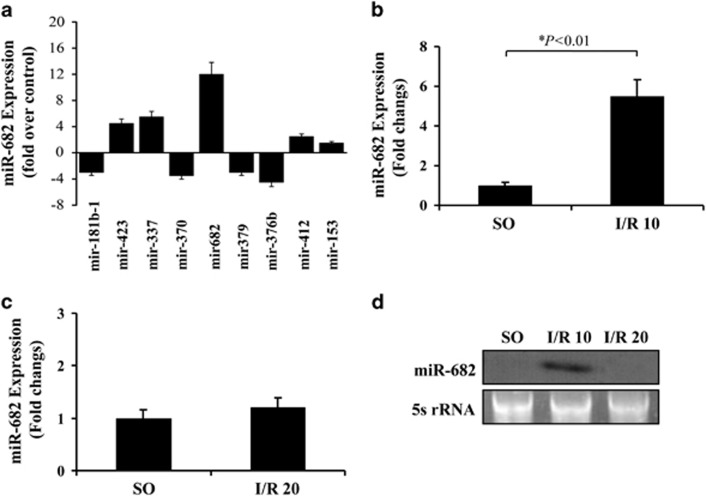
Upregulation of miR-682 in IEC of mice during intestinal ischemia. (**a**) Significant alteration in miRNA expression in IEC of mice during intestinal ischemia. Mice were subjected to 1 h of the superior mesenteric artery ischemia, while control mice had sham operation (SO). Values are means±S.D., *n*=10 in each group. Four independent experiments were performed. (**b**) Real-time PCR of miR-682. RNA from IEC isolated from sham-operated control mice or mice with ischemia following by 10:min of reperfusion (I/R 10). Values are means±S.D., *n*=6 in each group. Three independent experiments were performed. (**c**) Real-time PCR of miR-682. RNA from IEC isolated from sham-operated control mice or mice with ischemia following by 20 min of reperfusion (I/R 20). Values are means±S.D., *n*=6 in each group. Three independent experiments were performed. (**d**) Northern blot analysis of miR-682. Ten micrograms of the total RNA extracted from IEC isolated from sham-operated control mice or mice with ischemia following by 10 or 20 min of reperfusion were used for Northern blot analysis. 5s r-RNA was probed as loading control

With real-time PCR, total RNA samples extracted from isolated IECs of mice after intestinal ischemia were used for detecting the altered miRNAs expression. Of ~30 miRNAs analyzed which were chosen from the expression profile of miRNAs in mouse embryos,^23^ only 9 of miRNAs showed stable and significant changes in expression following intestinal ischemia. While miRNAs 181b-1, 370, 379, 376b showed a decrease, miRNAs 423, 337, 682, 412, 153 were upregulated (Figure 1a). Among these miRNAs, miR-682 showed an unvarying and highest upregulation after intestinal ischemia, but was undetected rapidly following 20 min of intestinal reperfusion (Figures 1b and c). miR-682 induction during intestinal I/R injury was confirmed by Northern analysis (Figure 1d). miR-682 was previously found via the massively parallel signature sequencing technology, but little is known about its targets and function. Next, we will explore the role and significance of miR-682 in the IEC of mice following intestinal I/R injury.

### HIF-1*α* mediates miR-682 induction during hypoxia

**Figure 2:**
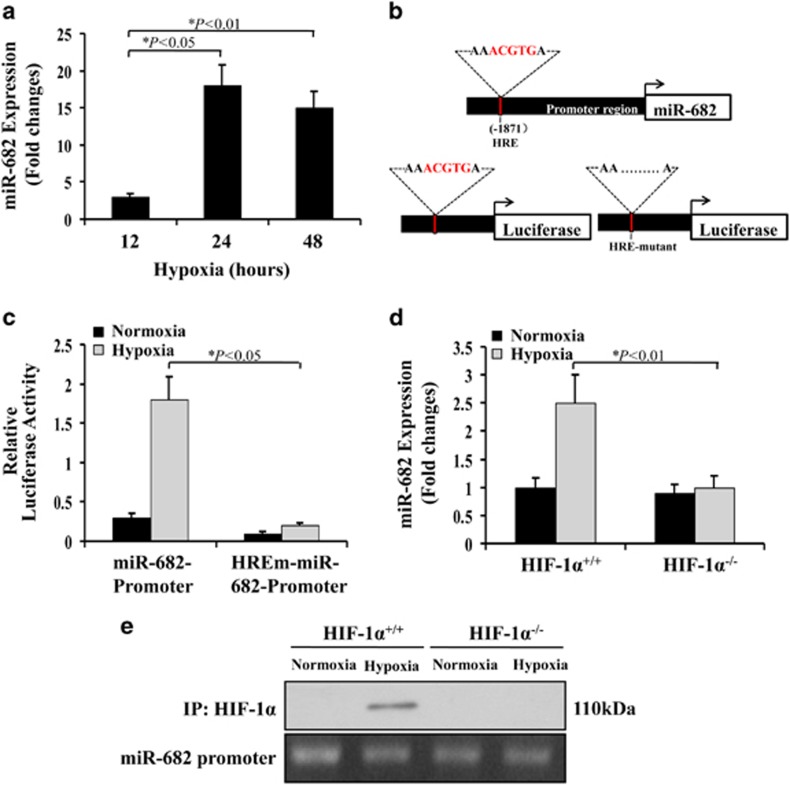
HIF-1 mediates miR-682 induction during hypoxia. (**a**) Induction of miR-682 by hypoxia. CCC-HIE-2 cells were incubated under hypoxia (1% oxygen) for 12–48 h to extract RNA for real-time PCR analysis of miR-682. Fold changes relative to cells without hypoxia treatment. Three independent experiments were performed. (**b**) Upper: miR-682 promoter region harboring HRE- or HIF-binding site. Lower: promoter reporter vectors containing the miR-682 promoter with or without HRE upstream of luciferase gene. (**c**) Activation of miR-682 promoter by hypoxia. miR-682 promoter or its HIF-binding site-deletion mutant were subcloned upstream of the luciferase gene in the promoter reporter construct. CCC-HIE-2 cells were contransfected with one of these reporter constructs, along with the Renilla luciferase construct, in a ratio of 2:0.1 and were then subjected to 24 h of hypoxia to collect lysate to measure luciferase activities. Four independent experiments were performed. (**d**) Induction of miR-682 expression by hypoxia in HIF-1*α*^+/+^ MEFs, but not in HIF-1*α*^−/−^cells. The cells were subjected to 24 h of hypoxia to isolated RNA for real-time PCR analysis of miR-682. Four independent experiments were performed. (**e**) HIF-1 binding to miR-682 promoter during hypoxia. HIF-1*α*^+/+^ and HIF-1*α*^−/−^ cells were incubated under hypoxia or normoxia for 24 h. Cell lysate was collected for ChIP analysis of HIF-1 binding to miR-682 promoter DNA. Three independent experiments were performed. MEF, mouse embryonic fibroblast

**Figure 3:**
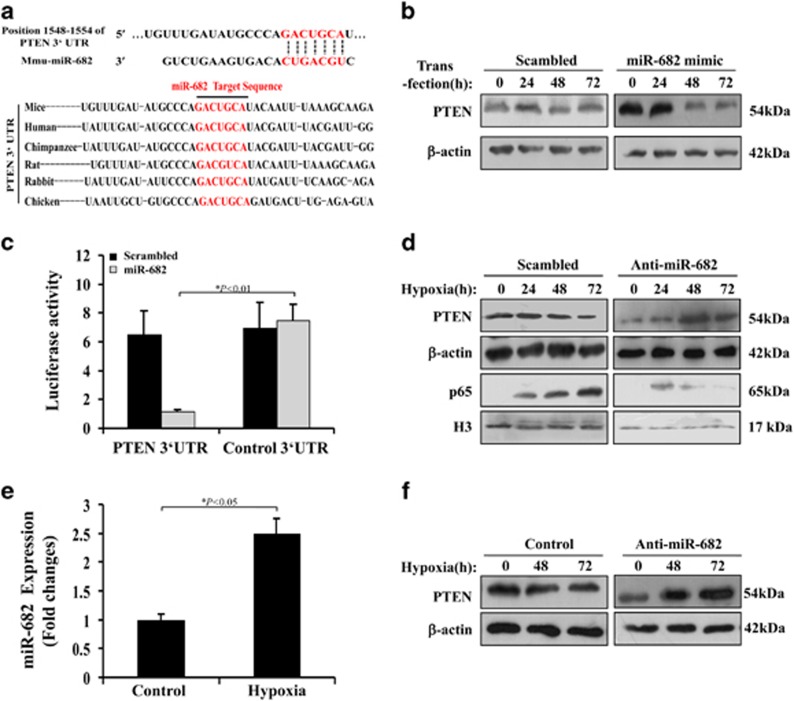
miR-682 targets PTEN during hypoxic condition. (**a**) Upper: putative miR-682 complementary sequence in the 3′UTR of murine PTEN mRNA. Lower: conserved miR-682 target sequence in the PTEN 3′UTR. (**b**) CCC-HIE-2 cells transfected with a control Scrambled or miR-682 mimic, and western blot analysis was carried out from the whole cell lysates collected at different time points. Four independent experiments were performed. (**c**) Luciferase reporter assay was conducted using constructs with the PTEN 3′UTR or an antisense control sequence. CCC-HIE-2 cells were cotransfected with these constructs along with the scrambled or miR-682 mimic. Four independent experiments were performed. (**d**) CCC-HIE-2 cells transfected with a control Scrambled or anti-miR-682 LNA were subjected to hypoxia, and whole cell lysates were collected at indicated time points. Four independent experiments were performed. (**e**) Quantitative PCR analysis of miR-682 gene expression in CCC-HIE-2 cells treated under hypoxia condition after 12 h. (**f**) IEC-6 cells transfected with a control scrambled LNA or anti-miR-682 LNA were subjected to hypoxia, and whole cell lysates were collected at indicated time points. Western blot analysis of these lysates. Four independent experiments were performed

Hypoxia-inducible factor-1 (HIF-1*α*) has been found to be the 'master' regulator of gene expression during hypoxia or ischemia of tissues and organs, including intestines.^24, 25^ The relationship between HIF-1*α* and miR-682 induction during hypoxia will be investigated. Hypoxia (1% oxygen) *in vitro* induced miR-682 in both human embryonic intestinal mucosa derived cells (CCC-HIE-2) and rat small intestine (IEC-6) cells (Figures 2a and 3e; Supplementary Figure 1E). Based on sequence analysis in databases (UCSC, JASPAR), the miR-682 promoter region of ~0.2 kb and its putative hypoxia responsive element (HRE)-deletion mutant (HIF-1-binding site) were cloned into luciferase promoter reporter vector (Figure 2b), which were then transfected into CCC-HIE-2 cells to determine their promoter activity. Hypoxia incurred a twofold increase in the activity of miR-682 promoter but not in the HRE-deletion mutant (HREm) promoter sequence (Figure 2c). The putative HIF-1-binding site of miR-682 promoter may have facilitated miR-682 upregulation under hypoxia condition. To anatomize whether miR-682 induction relies on HIF-1, wild-type and HIF-1*α*-null mouse embryonic fibroblasts were subjected to hypoxia to analyze miR-682 expression. We found that miR-682 was markedly induced by hypoxia in wild-type cells but not in HIF-1*α*-null cell (Figure 2d). By inhibiting HIF-1*α* expression, we also found that miR-682 expression was not induced in 72 h under hypoxia (Supplementary Figure 1F). Moreover, chromatin immunoprecipitation (ChIP) analysis determined the binding of HIF-1*α* on the miR-682 promoter region in hypoxic cells (Figure 2e). These data suggest that HIF-1*α* is the critical factor in miR-682 induction under hypoxic condition.

### miR-682 targets PTEN during hypoxia

We next analyzed the downstream gene targets of miR-682. Using database, target prediction (e.g., Targetscan, Boston, MA, USA) indicated that PTEN seemed as a candidate of putative gene targets for miR-682. A conserved, potential targeting site of miR-682 was found in the 3′ untranslated region (3′ UTR) of PTEN mRNA in various animal species (Figure 3a). First, we proved that miR-682 mimic transfected significantly induced miR-682 expression in CCC-HIE-2 cells (Supplementary Figure 1A). Transfection of miR-682 mimic, but not scrambled sequence, alleviated PTEN expression in CCC-HIE-2 cells after 48 h (Figure 3b and Supplementary Figure 2A). We further investigated the effect of miR-682 on the 3′ UTR of PTEN using miRNA Target Luciferase Reporter system. PTEN 3′ UTR and its reverse sequence (control 3′ UTR) were cloned downstream of the luciferase reporter gene driven by a constitutive promoter. The constructs were transfected into CCC-HIE-2 cells along with miR-682 mimic or a sequence-scrambled oligonucleotide. We found that miR-682 mimic significantly reduced luciferase expression in luciferase-PTEN 3′ UTR-transfected cells, while the sequence-scrambled had no effective (Figure 3c). Expectedly, neither sequences significantly changed luciferase expression in luciferase-control 3′ UTR-transfected cells. Moreover, using site-directed mutagenesis to construct PTEN 3′ UTR mutant by mutating the miR-682-targeted sequence, luciferase reporter experiment was performed again (Supplementary Figures 1B and C) and further support that miR-682 directly inhibit PTEN expression. However, In CCC-HIE-2 cells, hypoxia induced a decrease in PTEN expression and a increase of nuclear translocation of p65 at 48 h after hypoxia, and the decrease was reversed and the increase was counteracted by anti-miR-682 (Figure 3d and Supplementary Figure 2B). This can explain that apoptotic index was significantly raised by anti-miR-682 treatment (Supplementary Figure 1G). Anti-miR-682 transfected was proved to be downregulated miR-682 expression induced by hypoxia (Supplementary Figure 1D). Similarly, in IEC-6 cells, miR-682 expression was on a rise during hypoxia and anti-miR-682 transfection promoted PTEN expression under hypoxia (Figures 3e and f and Supplementary Figure 2C). These strongly imply that miR-682 directly target PTEN expression under normoxia and hypoxic condition.

### miR-682 protects small intestine against I/R injury through PTEN/NF-*κ*B p65 pathway

**Figure 4:**
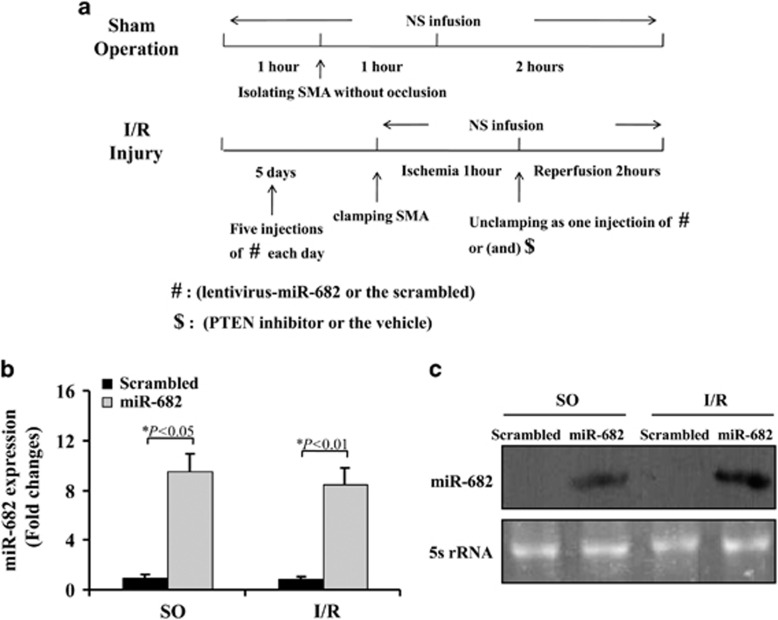
miR-682 protects small intestine against ischemia–reperfusion injury through PTEN/NF-*κ*B p65 pathway. (**a**) The protocol of experiments. Sham-operated (SO) group: involving isolation of the superior mesenteric artery (SMA) without occlusion; ischemia–reperfusion Injury (I/R) group and treatments: performed by 1 h occlusion of SMA followed by 2 h of reperfusion without intervention. (**b**) Quantitative PCR analysis of miR-682 gene expression in isolated IEC cells of mice treated with or without I/R injury with a scrambled control or lentivirus-miR-682 (miR-682). Ten independent experiments were performed. (**c**) Northern blot analysis of miR-682 expression of IEC of mice after six i.v. injections of lentivirus-miR-682 (miR-682) with or without I/R injury. Forty micrograms of the total RNA extracted from isolated IEC were used for Northern blot analysis. 5 s r-RNA was probed as loading control. Six independent experiments were performed

**Figure 5:**
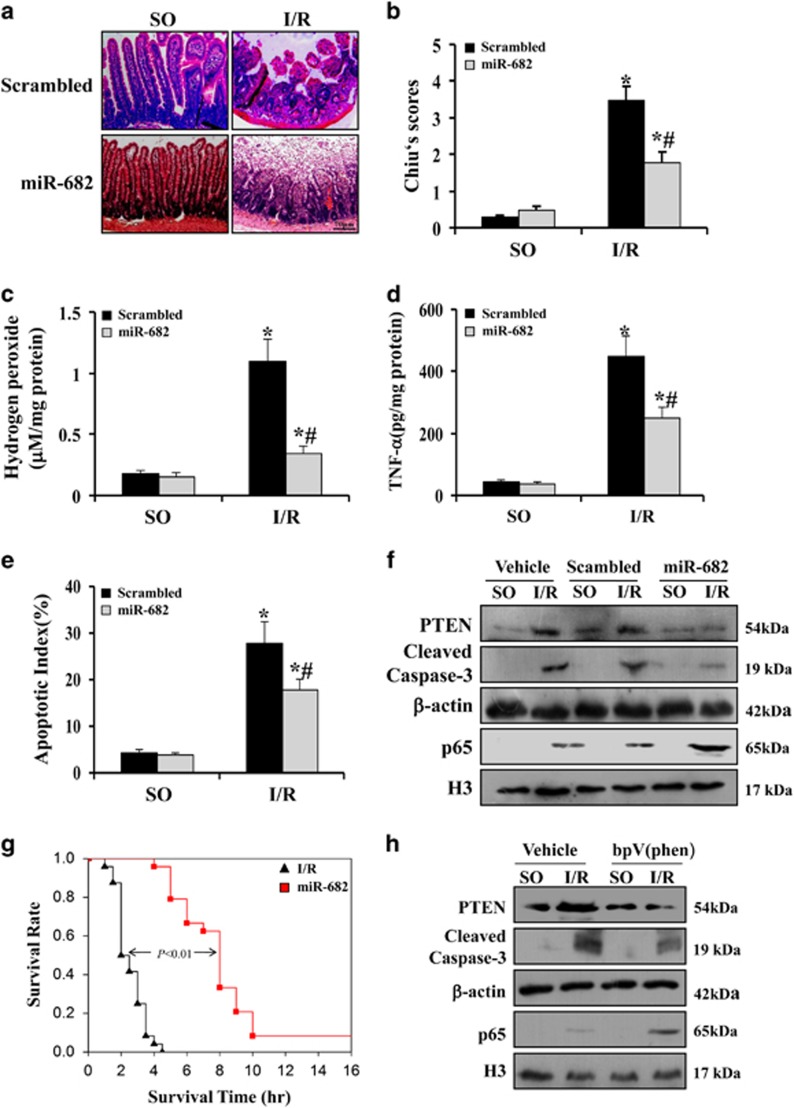
miR-682 protects small intestine against ischemia–reperfusion injury through PTEN/NF-*κ*B p65 pathway. (**a**) H&E staining was performed using formalin-fixed tissue sections from sham-operated control (SO) mice or (I/R) mice with 60 min of ischemia following by 120 min of reperfusion with or without pretreatment with lentivirus-miR-682 (miR-682) or a scrambled control. Magnification: × 400. (**b**) Chiu′s scores were measured and compared by ANOVA with Tukey post test. *P<0.05 versus SO. ^**#**^*P*<0.05 versus Scrambled. Values are means±S.D., *n*=6 in each group. Three independent experiments were performed. (**c**) Levels of hydrogen peroxide were measured in IEC isolated from SO mice and I/R mice with or without pretreatment of lentivirus-miR-682 (miR-682) or a scrambled control. *P<0.01 versus SO. ^**#**^*P*<0.05 versus Scrambled. Values are means±S.D., *n*=6 in each group. Three independent experiments were performed. (**d**) ELISA analysis of TNF-*α* protein expression in the IEC isolated from SO mice and I/R mice with or without pretreatment of lentivirus-miR-682 (miR-682) or a scrambled control. *P<0.01 versus SO. ^**#**^*P*<0.01 versus Scrambled. Values are means±S.D., *n*=6 in each group. Three independent experiments were performed. (**e**) Apoptotic index was measured by counting a minimum of 20 randomly selected villi and crypts in the sections following TUNEL staining. The index was obtained by dividing the TUNEL-positive cells by the total number of cells. *P<0.05 versus SO. ^**#**^*P*<0.05 versus Scrambled. Values are means±S.D., *n*=6 in each group. Three independent experiments were performed. (**f**) Western blot analysis of the IEC isolated from SO mice and I/R mice with or without pretreatment of lentivirus-miR-682 (miR-682) or a scrambled control. *β*-actin and nuclear H3 were used as the controls for loading. (**g**) Survival curves of I/R mice with or without pretreatment of lentivirus-miR-682 (miR-682). Values are means±S.D., *n*=8 in each group. Three independent experiments were performed. (**h**) Western blot analysis of the IEC isolated from SO mice and I/R mice with or without pretreatment of PTEN inhibitor bpV(phen) or vehicle. *β*-actin and nuclear H3 were used as the controls for loading

To study the *in vivo* role of miR-682 in intestinal I/R injury, we used systemic injection of lentivirus-miR-682 to overexpress miR-682 in IEC of mice. Based on the protocol of experiment (Figure 4a), miR-682 was significantly detected in IEC of mice after systemic injection of lentivirus-miR-682 (Figures 4b and c). First, intestinal mucosal morphology was evaluated by using Chiu's score. The mice receiving miR-682 showed significantly less intestinal injury than those with a control, sequence-scrambled (scrambled) (Figures 5a and b), and meanwhile, it also showed that oxidative stress and inflammatory response were greatly reduced, compared with mice treated with the scrambled (Figures 5c and d). Given that oxidative stress and inflammation have closely relationship with cell apoptosis, the terminal deoxynucleotidyl transferase-mediated dUTP–biotin nick end-labeling assay (TUNEL) was performed. The finding revealed that decreased apoptotic index was observed in mice with miR-682 (Figure 5e). With analysis of molecular biology, we found that I/R-induced PTEN expression and caspase-3 activity were markedly reduced, but nuclear translocation of NF-*κ*B p65 was rapidly augmented in the IEC of mice with miR-682, compared with mice treated with scrambled (Figure 5f and Supplementary Figures 3A–C). The morphological and molecular change in mice with or without miR-682 following I/R injury could explain that miR-682 significantly prolonged survival in mice in response to I/R (Figure 5g). Finally, to further determine the importance of PTEN on intestinal I/R injury, PTEN inhibitor was used and found that caspase-3 activity was reduced and nuclear shift of NF-*κ*B p65 was elevated in mice receiving PTEN inhibitor following I/R injury (Figure 5h and Supplementary Figure 3D–F). These results suggest that miR-682-mediated downregulation of PTEN is a critical factor in the initiation and development of intestinal I/R injury and exert its effect in activating NF-*κ*B pathway.

### miR-682 suppresses hypoxia-induced cell apoptosis via PTEN/NF-*κ*B p65 pathway

**Figure 6:**
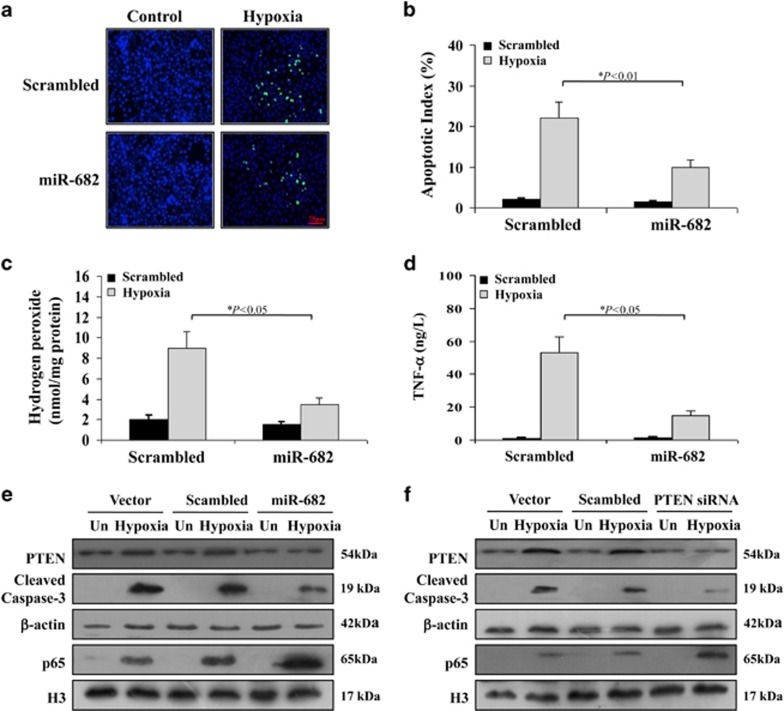
miR-682 suppresses hypoxia-induced cell apoptosis via PTEN/NF-*κ*B p65 pathway. (**a**) TUNEL fluorescent staining in CCC-HIE-2 cells subjected to hypoxia after 24 h, with or without pretreatment with miR-682 mimic or a scrambled control. Magnification: × 400. (**b**) The apoptotic index was calculated by counting a minimum of 20 randomly selected fields following TUNEL staining. The index was obtained by dividing the TUNEL-positive cells by the total number of cells. Values are means±S.D.. Three independent experiments were performed. (**c**) Levels of hydrogen peroxide were measured in CCC-HIE-2 cells under hypoxia conditions after 24 h with or without pretreatment of miR-682 mimic or a scrambled control. Three independent experiments were performed. (**d**) ELISA analysis of TNF-*α* protein expression in CCC-HIE-2 cells under hypoxia conditions after 24 h with or without pretreatment of miR-682 mimic or a scrambled control. Three independent experiments were performed. (**e**) Western blot analysis of CCC-HIE-2 cells subjected to hypoxia after 24 h with or without pretreatment of miR-682 mimic or a scrambled control. *β*-actin nuclear H3 was used as the control for loading. Three independent experiments were performed. (**f**) Western blot analysis of CCC-HIE-2 cells subjected to hypoxia after 24 h with or without pretreatment of PTEN siRNA or a scrambled control. *β*-actin nuclear H3 was used as the control for loading. Three independent experiments were performed

The previous evidences have demonstrated that miR-682 has a protective influence in intestinal I/R injury through regulating PTEN/NF-*κ*B p65 pathway *in vivo*. We further determined the effect of miR-682 *in vitro* under hypoxia condition. Hypoxia markedly induced cell apoptosis, which was blocked by 50% in cells with miR-682 treatment (Figures 6a and b). Oxidative stress and inflammatory factor tumor necrosis factor-*α* (TNF-*α*) under hypoxia were highly expressed, but could be prohibited by miR-682 treatment (Figures 6c and d). What's more, miR-682 greatly inhibited PTEN expression and caspase-3 activity induced by hypoxia, and facilitated nuclear shift of NF-*κ*B p65 which showed it could defend against intestinal I/R injury^26^ to prevent cell apoptosis (Figure 6e and Supplementary Figures 4A–C). Last, we used PTEN siRNA to investigate *in vitro* the effect of PTEN under hypoxia and found that caspase-3 activity was reduced while nuclear shift of NF-*κ*B p65 was elevated (Figure 6f and Supplementary Figures 4D–F). These results indicate that miR-682 lessens oxidative stress, inflammatory reaction and cell apoptosis via PTEN/ NF-*κ*B p65 pathway.

### miR-682 represses mitochondrial-mediated apoptosis in IEC of mice in response to intestinal I/R injury

**Figure 7:**
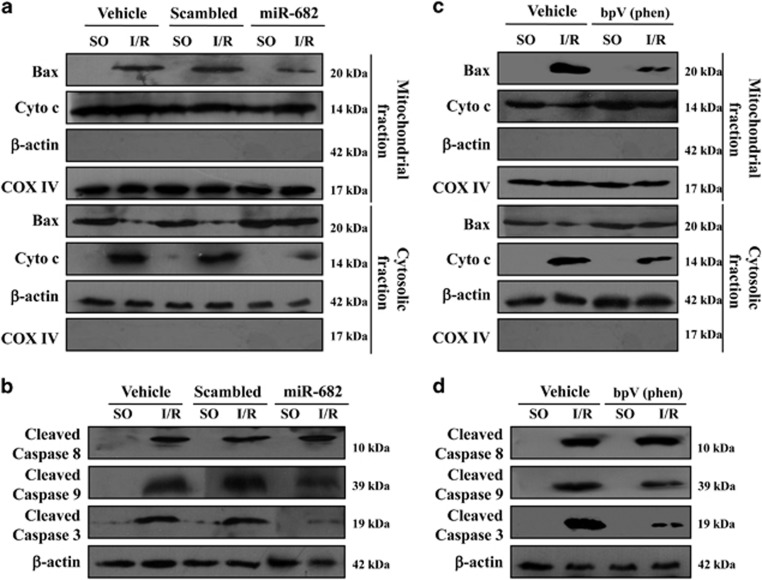
miR-682 represses mitochondrial-mediated apoptosis in IEC of mice in response to intestinal I/R injury (**a**) Mitochondrial and cytosolic fractions were analyzed for Bax and cytochrome *c* by western blotting. *β*-actin and COX IV were markers of cytosolic and mitochondrial fractions, respectively. Three independent experiments were performed. (**b**) Western blot analysis of caspase-8, -9 and -3 activity of IEC cells isolated from sham-operated control (SO) mice or (I/R) mice with or without pretreatment with lentivirus-miR-682 (miR-682) or a scrambled control. (**c**) Mitochondrial and cytosolic fractions were analyzed for Bax and cytochrome *c* by western blotting. *β*-actin and COX IV were markers of cytosolic and mitochondrial fractions, respectively. Three independent experiments were performed. (**d**) Western blot analysis of caspase-8, -9 and -3 activity of IEC from sham-operated control (SO) mice or (I/R) mice with or without pretreatment with PTEN inhibitor bpV(phen) or vehicle

To investigate the mechanism of miR-682-mediated anti-apoptosis following I/R injury, we analyzed several mitochondria-related events in IEC of mice. In the sham-operated mice with or without vehicle or scrambled, Bax was detected in the cytosolic fractions, but not in the mitochondrial fractions and cytochrome *c* was measured in the michondrial fractions, but not in the cytosolic fractions (Figure 7a and Supplementary Figures 5A and B). However, the level of Bax was markedly increased in the mitochondrial fractions and the release of cytochrome *c* was on a significant rise in the cytosolic fractions in IEC of mice in response to I/R injury. I/R injury also induced caspase-3 and -9 activity in IEC of mice following I/R injury. More importantly, miR-682 remarkably alleviated mitochondrial translocation, the release of cytochrome *c* and caspase-3 and -9 activity in IEC of mice after intestinal I/R injury (Figures 7a and b and Supplementary Figures 5E and F). The previous evidence above showed that PTEN is a key target of miR-682 in intestinal I/R injury. To detect PTEN as a critical mediator of I/R-induced mitochondrial-mediated apoptosis, PTEN inhibitor was used and found that mitochondrial translocation, the release of cytochrome *c* and caspase-3 and -9 activity in IEC of mice following I/R injury were distinctly diminished (Figures 7c and d and Supplementary Figures 5C,D,G and H). Collectively, these strongly prove that miR-682 prevents I/R-induced apoptosis in IEC of mice via regulating mitochondrial pathway.

### NF-*κ*B inhibitor compromises intestinal protection of miR-682 following intestinal I/R injury

**Figure 8:**
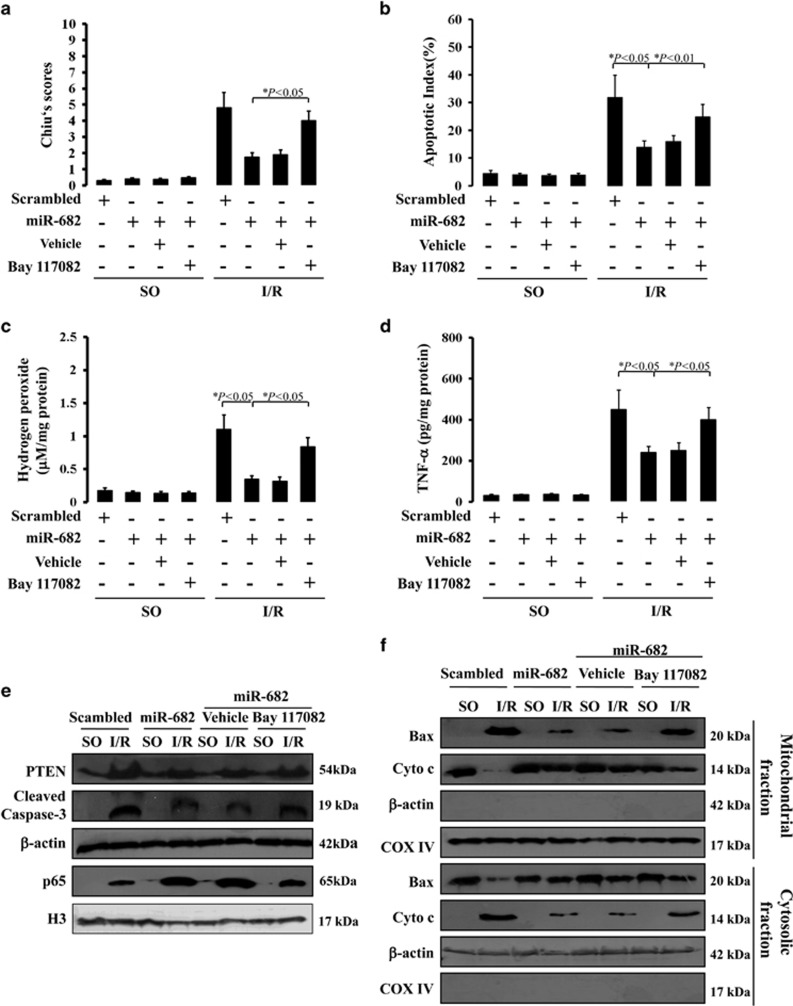
NF-*κ*B inhibitor compromises intestinal protection of miR-682 following intestinal I/R injury. (**a**) Chiu′s scores were measured and compared by ANOVA with Tukey post test. Values are means±S.D., *n*=6 in each group. Three independent experiments were performed. (**b**) Apoptotic index in the crypts measured by TUNEL staining. Values are means±S.D., *n*=6 in each group. Three independent experiments were performed. (**c**) Levels of hydrogen peroxide were measured in IEC isolated from SO mice and I/R mice with pretreatment of lentivirus-miR-682 (miR-682) or a scrambled control with or without treatment of Bay117082. Values are means±S.D., *n*=6 in each group. Three independent experiments were performed. (**d**) ELISA analysis of TNF-*α* protein expression in IEC isolated from SO mice and I/R mice with pretreatment of lentivirus-miR-682 (miR-682) or a scrambled control with or without treatment of Bay117082. Values are means±S.D., *n*=6 in each group. Three independent experiments were performed. (**e**) Western blot analysis of IEC isolated from SO mice and I/R mice with pretreatment of lentivirus-miR-682 (miR-682) or a scrambled control with or without treatment of Bay117082. *β*-actin and nuclear H3 were used as the controls for loading. Three independent experiments were performed. (**f**) Western blot analysis of mitochondrial and cytosolic fractions from IEC isolated from SO mice and I/R mice with pretreatment of lentivirus-miR-682 (miR-682) or a scrambled control with or without treatment of Bay117082. *β*-actin and COX IV were markers of cytosolic and mitochondrial fractions, respectively. Three independent experiments were performed

Our previous data showed that NF-*κ*B p65 is an important downstream target regulated by miR-682-mediated PTEN expression following intestinal I/R injury. To further confirm the effect of NF-*κ*B pathway on intestinal I/R injury and miR-682-mediated PTEN expression, administration of the NF-*κ*B inhibitor, Bay117082, was used. Histologic measurement of intestinal mucosal injury was assessed in scrambled-treated control mice or lentivirus-miR-682-treated mice with or without Bay117082. NF-*κ*B inhibitor could significantly neutralize the intestinal morphological protection by miR-682 treatment in intestinal I/R injury (Figure 8a). With regard to intestinal I/R-induced apoptosis, enhanced apoptotic index was observed in miR-682-treated mice with Bay117082, compared with lentivirus-miR-682-treated mice (Figure 8b). Moreover, NF-*κ*B inhibitor also impaired intestinal benefit of miR-682 treatment on oxidative stress and inflammatory factor induced by I/R injury. Hydrogen peroxide expression and TNF-*α* expression in IEC was greatly re-elevated in lentivirus-miR-682-treated mice after Bay117082 treatment (Figures 8c and d). Next, we found that NF-*κ*B inhibitor could sharply promoted caspase-3 activity and mitochondrial-mediated apoptotic events, but did not change the PTEN expression in IEC of lentivirus-miR-682-treated mice with Bay117082 treatment following I/R injury, compared with control mice (Figures 8e and F and Supplementary Figures 6A–E). Furthermore, analysis of mitochondrial-related events showed IEC of lentivirus-miR-682-treated mice with Bay117082 had more serious mitochondrial damage than that of lentivirus miR-682-treated mice. These events suggest that the NF-*κ*B pathway has a protective impact on intestinal I/R injury and is downstream of miR-682-mediated PTEN expression.

## Discussion

This study has identified a miRNA-mediated signaling pathway that contributes to hypoxic and ischemic injury in intestine epithelial cells and tissues. We showed miR-682 is a critical mediator in preventing IEC apoptosis induced by I/R mainly via modulation of PTEN expression, subsequent inactivation of NF-*κ*B pathway. miR-682 attenuated intestinal disintegrity and prolonged the survival of mice following intestinal I/R. To our knowledge, there are few reports in which single miRNA significantly protests against intestinal I/R injury by inhibiting IEC apoptosis.

To study the role of miR-682 in intestinal I/R injury *in vivo*, we used systemic injection of lentivirus-miR-682 to overexpress miR-682 in intestines (Figure 4). miR-682 significantly prohibited cell apoptosis during hypoxia and intestinal I/R, suggesting that miR-682 is anti-apoptotic. Consistently, anti-miR-682 facilitated apoptosis in hypoxic IEC-6 cells. Mechanically, The target of miR-682 on PTEN is directly responsible for the anti-apoptotic effect. PTEN is involved in multiple regulation by miRNAs. In intestines, several miRNAs modulate PTEN, resulting in pathophysiologic alterations. miR-21 and miR-26 have been demonstrated that these could directly regulate PTEN expression in intestinal neoplasms.^27, 28^ Our current work has further proved that miR-682 has direct effect on PTEN for upregulation under conditions of hypoxia and ischemia in intestine cells and tissues. PTEN is generally known to repress cell survival signaling, such as Akt, and therefore promotes cell death.^29^ In our experiments, changes in PTEN expression following overexpression or silencing of miR-682 could not affect the expression of AKT, suggesting AKT may be not involved in miR-682/PTEN pathway following intestinal I/R. Further work will explain it.

NF-*κ*Bs which are a family of transcription factors, have an important function in regulating cell anti-apoptosis, cell proliferation, inflammation and immune response. Signaling cascades induced by LPS, IL-1, TNF*α* or DNA double strands breaks activate the I*κ*B (inhibitor of *κ*B)–kinase (IKK) complex, which phosphorylates I*κ*B proteins leading to their degradation. The series of the signal transduction transfer the accumulated dissociative NF-*κ*B in cytoplasm into the nucleus and intranuclear *κ*B bind to the promoter regions of specific target genes involving encoding anti-apoptotic and pro-apoptotic proteins.^30^ In this study, we showed intestinal NF-*κ*B activation by miR-682 following I/R injury could protect the IECs from apoptosis, as consistent as that gut I/R in mice lacking IKK-*β* in enterocytes resulted in considerable tissue damage and a marked increase in the apoptotic index.^31^ Conversely, some indicate that inhibiting NF-*κ*B activation as a promising molecular target for ameliorating reperfusion injury in the brain during stroke, the heart during myocardial infarction and the intestines following prolonged ischemia.^32, 33, 34, 35^ The disparity between our findings and those might be partly explained by I/R injury in different organs of mice, ischemia time or magnitude of inflammation. Previous study also showed that gut I/R resulted in a marked increase in serum TNF-*α* and accumulation of TNF-*α* in the mucosa, both of which are prevented by ablating IKK-*β* in enterocytes. However, in this study, NF-*κ*B activation by miR-682 prohibited the increase of TNF-*α* in the mucosa. The main reason to explain is the different ischemia time with 60 min in this study and 30 min in the other studies. Future works need to provide more details on a crucial function of NF-*κ*B pathway in regulating inflammation and apoptosis in response to intestinal I/R injury.

In conclusion, this study has emphasized miR-682 as an important miRNA in preventing intestinal I/R injury. The elaboration of the miR-682/PTEN/NF-*κ*B signaling pathway not only provides novel and important insights into the pathogenesis of intestinal I/R injury but also suggests a new miRNA-based therapeutic target for prevention and treatment.

## Materials and Methods

### Animals, surgery and treatments

The current study was approved by the Animal Care and Use Committee of Sun Yat-sen University, Guangzhou, China. C57/BL6 mice (20–25 g) were anaesthetized by i.p. injection of 4% chloral hydrate (200 mg/kg) during intestinal ischemia. A laparotomy was carried out under chloral hydras anesthesia, and the superior mesenteric artery was occluded with a micro-bulldog clamp suitable for mice.^36^ The surgical procedure was performed on a heated-table thermostated at 38 °C appropriate. After 60 min, the clamp was removed, the abdominal skin sutured and the tissue was subjected to reperfusion for 10 or 20 or 120 min. The mice were then killed.

The intestinal ischemic/reperfusion tissue was carefully removed, placed on ice and was rinsed thoroughly with physiological saline and then cut into ~2 cm sections in length. Some were fixed in 10% neutral buffered formalin for measurement of histological analysis and terminal TUNEL assay. Some were opened longitudinally on anti-mesenteric border to expose the intestinal mucosa. IECs were isolated as described below.

Lentivirus-miR-682 was produced as previously described.^37^ Lentivirus encoding shRNAs for miR-682 was purchased (GenePharma, Shanghai, China). The sequence of miR-682 is 5′-CTGCAGTCACAGTGAAGTCTG-3′ and the sequence of the scrambled as the negative control is 5′-CAGACTTCACTGTGACTGCAG-3′, were subcloned to pLV/H1/GFP vectors. To produce lentivirus containing miR-682, HEK-293T cells (American Type Culture Collection (ATCC, Rockefeller, MD, USA)) were cotransfected with pLV-miR-682 plasmid and ViraPower Packaging Mix using Lipofectamine 2000 (Invitrogen, Carlsbad, CA, USA). Lentivirus (10^9^ TU/ml) or its control (10^9^ TU/ml) was mixed with the cationic lipid polybrene (4 *μ*g/*μ*l, GenePharma) and incubated at 37 °C for 15 min.

#### Treatments

mice received six i.v. injections of Lentivirus-miR-682 or its control scrambled sequence. Five injections were given daily for 5 consecutive days before intestinal ischemia and the last injection was given at the beginning of reperfusion. Overexpression of miR-682 in IECs of mice was investigated. In addition, some mice were also injected i.v. with optimum dose of 0.4* μ*mol PTEN inhibitor bpV(phen) or 20 mg/kg NF-*κ*B inhibitor Bay117082 (both from Sigma Chemical Co, St Louis, Missouri, USA) or delivered at the beginning of reperfusion.

### Isolation of IECs

The small IECs were isolated as previously described.^38^ Briefly, isolated small intestines were opened longitudinally, and washed with cold PBS. The tissue was chopped into ~5 mm pieces, and further washed with cold PBS. The tissue fragments were incubated in 2 mM EDTA with PBS for 30 min on ice. After removal of EDTA medium, the tissue fragments were vigorously suspended by using a 10- ml pipette with cold PBS. This fraction was passed through a 70-mm cell strainer (BD Bioscience, Franklin Lake, NJ, USA) to remove residual villous material. Isolated cells were collected for PCR, western blotting, Bax and cytochrome *c* translocation.

### Total RNA extraction and real-time PCR

Total RNA was extracted from the IECs isolated from small intestines of mice using the RNAgents Total RNA Isolation System (Promega, Madison, WI, USA) according to the manufacturer's instructions. About 40 ng of total RNA was reverse-transcribed into cDNA by using the miRNA Reverse Transcription kit (Applied Biosystems, Foster City, CA, USA). The TaqMan microRNA Assay (Qiagen, Hilden, Germany) was used for real-time PCR using sequence-specific primers for cDNA synthesis and Taqman probes for real-time PCR. Fold change in expression were quantified using ΔCt values. About 30 novel miRNAs chosen from the expression profile of miRNAs in mouse embryos^23^ were analyzed. Their sequences are described in Table 1.

### Cell culture, hypoxia, treatments and apoptosis

Human embryonic intestinal mucosa derived cell line CCC-HIE-2 was originally obtained from China Infrastructure of Cell Line Resources (Chinese Academy of Medical Sciences) and IEC-6 cell line was from ATCC and were cultured in complete medium consisting of DMEM (CCC-HIE-2) or DMEM/F12 (IEC-6) (Thermo Fisher Scientific, Waltham, MA, USA) supplemented with 10% fetal bovine serum at 37 °C in CO_2_ incubator. For hypoxia treatment, overnight cultured cells were transferred into hypoxia chamber with 1% oxygen and incubated in pre-equilibrated medium for indicated time periods. Under hypoxia condition after 24 h, cells were collected for protein extraction.

miR-682 mimic (100 nmol/l) or its scrambled control (Invitrogen) and anti-miR-682 LNA (100 nmol/l) or its scrambled control (Exiqon, Vedbaek, Denmark) were used *in vitro* experiments. They were transfected by using Lipofectamine 2000 into CCC-HIE-2 or IEC-6 cells for 48 h and used in hypoxia experiments.

PTEN knockdown was achieved by using PTEN siRNA (Santa Cruz Biotechnology, Santa Cruz, CA, USA) according to the manufacturer's instructions. After adding the mixture, containing Lipofectamine and PTEN siRNA dissolved in Optimal (Invitrogen), to cells of 80% confluence and at 48 h after interference, cells were exposed to hypoxia treatment and harvested. Fluorescein conjugated control siRNA was used for indication of successfully transfection into cells.

Intestinal cell apoptosis was evaluated by using an *in situ* cell death detection Kit (Roche, Basel, Swiss), according to the manufacturer's instruction. With TUNEL staining, the apoptotic index was microscopically determined by dividing the number of apoptotic cells by the total number of cells in glass slide of at least 20 randomly selected fields (× 400).

### Histological and intestinal TUNEL analysis

The segment of small intestine was stained with hematoxylin and eosin. Damage of intestinal mucosa was evaluated using criteria of Chiu′ s method^39^ by two independent experienced pathologists who were blinded to the study groups. A minimum of six randomly chosen fields from each mouse were evaluated and averaged to determine mucosal damage, and the results of two pathologists were averaged.

TUNEL staining was carried out using an *in situ* cell death detection kit according to the manufacturer's instructions. Apoptotic index was measured by counting a minimum of 20 randomly selected villi and crypts in the sections (× 400) following TUNEL staining. The index was obtained by dividing the TUNEL-positive cells by the total number of cells.^36^ The data was valued as mean±S.D. Three independent observers blinded to groups and treatments were responsible for apoptotic cells scoring.

### Northern blot analysis

Northern analysis of miRNA was conducted as described previously.^40^ Briefly, 10 *μ*g total RNA isolated with Ambion RNA extraction kit (Applied Biosystems) was resolved on 15% acrylamidebisacrylamide gel (19:1) containing 7 M urea in Tris Borate EDTA buffer. After transfer to Hybond membrane (Amersham, Uppsala, Sweden) and ultraviolet crosslinking, the membrane was incubated with the radiolabeled hybridization probe in Ultra-Hyb-oligo hybridization buffer (Ambion, Thermo Fisher Scientific, Waltham, MA, USA). The membrane was then washed extensively before exposure to X-ray films at −70 °C.

### ChIP assay

ChIP analysis of HIF-1*α* binding to miR-682 promoter was performed by using an assay kit (R&D Systems, Minneapolis, MN, USA), following manufacturer's instruction. Briefly, after fixation with formaldehyde, cell lysate was collected. The samples were then sonicated to shear chromatin for centrifugation and to collect supernatant for immunoprecipitation with anti-HIF-1*α* antibody. After washes, the resultant immunoprecipitates were subjected to PCR analysis using specific primers.

### Plasmid constructs and luciferase reporter assay

The human 3′-UTR of the *PTEN* gene was amplified by PCR using the primers (5′-CGATTCTAGAAATCATGTTCTGGTGG-3′) for PTEN-3′-UTR-Forward and (5′-GCATTCTAGAATTCTGCACAGTAAGCATA-3′) for PTEN-3′-UTR-Reverse. They were cloned into the *Xb*aI/*Xb*aI site of the pGL3 control vector (Promega) to generate the vector pGL3-PTEN. For the luciferase reporter assay, CCC-HIE-2 cells were cultured in 96-well plates and transfected with the pGL3-PTEN+miR-682 mimic, pGL3-PTEN, pGL3-control+miR-682 mimic or pGL3 control by using Lipofectamine 2000. At 48 h after transfection, the cells were collected, washed once with phosphate-buffered saline and analyzed with a Dual-Luciferase reporter assay system (Promega) according to the manufacturer's instructions with a Lumat LB 9507 luminometer (Berthold, Nashua, NH, USA).

### Site-directed mutagenesis

Site-directed mutagenesis was used to modify the miR-682 seed-binding sequence using Pfu Turbo DNA Polymerase (Merck, Darmstadt, Germany). *Dp*nI was used to digest non-mutated DNA template before transforming the mutated plasmids. The sequences of PTE′ 3′ UTR and PTEN 3′ UTR mutant were as follows: PTEN 3′ UTR: 3′-UAUUUGAUAUGCCCAGACUGCAUACGAUUUACGAUUGG-5′ The seven sequences in blue are miR-682 seed-binding sequences. PTEN 3′ UTR mutant: 3′-UAUUUGAUAUGCCCAGAUCGGAUACGAUUUACGAUUGG-5′. The sequences in red are the mutant sequences in miR-682 seed-binding sequence.

### Analysis of bax and cytochrome *c* translocation

To detect Bax and cytochrome *c* translocation, intestinal mucosal sample was used to isolate mitochondrial and cytosolic fractions by the differential centrifugation method as described previously.^36^ Briefly, after washed by ice cold PBS, the samples were resuspended in homogenization buffer (0.25 M sucrose, 10 mM HEPES, pH 7.4 and 1 mM EGTA). The homogenate was subjected to centrifugation at 1000  × *g* for 15 min at 4 °C to separate the nuclei and unbroken cells. The supernatant was subsequently centrifuged at 10 000 × *g* to harvest the cytosolic fraction (supernatant) and the mitochondrial fraction (pellet). The mitochondrial fraction was resuspended in homogenization buffer. Both fractions were analyzed by western blotting for Bax (Abcam, Cambridge, England).

### Nuclear extraction

Nuclear extraction was performed by Nuclear Extracts Kit (Active Motif, Carlsbad, CA, USA) according to manufacturer's instruction. The following procedures were based on samples of ~3.2 × 10^6^ cells. Before beginning, three buffers should be prepared. Buffer A was a 4 ml PBS/Phosphatase inhibitors buffer containing 0.4 ml 10 × PBS and 0.2 ml phosphatase inhibitors and 3.4 ddH_2_O, Buffer B was a 1x Hypotonic buffer and Buffer C was a Complete Lysis buffer consisting of 2.5 *μ*l 10 mM Dithiothreitol and 0.25 *μ*l protease inhibitor cocktail and 22.5 *μ*l lysis solution. The cells were washed three times with buffer A and centrifuged for 5 min at 500 r.p.m. in a centrifuge pre-cooled at 4 °C. Cell pellet at the bottom after 500 r.p.m. centrifuge was ready for cytoplasmic fraction collection. Resuspended in 500 *μ*l buffer B and incubated for 15 min on ice. About 25 *μ*l detergent was added before being centrifuged for 30 s at 14 000 ×  *g* in a centrifuge pre-cooled at 4 °C. Supernatant (cytoplasmic fraction) after 14 000 ×  *g* centrifuge was transferred into tub and stored at −80 °C. Nuclear pellet at the bottom after 14 000 ×  *g* centrifuge was ready for nuclear fraction collection. Resuspended in 50 *μ*l buffer C and incubated for 30 min on ice. Finally, centrifuged for 10 min at 14 000 ×  *g* in a centrifuge pre-cooled at 4 °C and supernatant (nuclear fraction) was transferred into tube and stored in −80 °C until ready to use for western blotting.

### TNF-*α* and hydrogen peroxide assays

The TNF-*α* concentration of IEC of mice was measured using a commercial kit (eBioscience, San Diego, CA, USA), according to the manufacturer's instruction. After the stop solution was added, the plates were read at 450 nm (570 nm correction) on a MicroPlate Reader (BioTek, Seattle, WA, USA). The values for results were expressed as pg TNF-*α*/mg protein.

Hydrogen peroxide assay was measured in IEC of mice by using the Amplex Red hydrogen peroxide/peroxidase assay kit (Invitrogen) according to the manufacturer's instructions. Absorbance at 560 nm and the fluorescence emission at 590 nm were measured to determine the level of hydrogen peroxide in a Victor III (Perkin Elmer/Wallace, Fremont, CA, USA) plate reader. The results of hydrogen peroxide were expressed as *μ*M hydrogen peroxide/mg total proteins.

### Western blotting and antibodies

About 50 *μ*g total proteins of small IECs was denatured in sample buffer containing SDS and *β*-mercaptoethanol, separated on a 4–20% gradient SDS-PAGE gel, and electroblotted onto nitrocellulose membranes. Nonspecific binding sites of the membrane were blocked using defatted milk protein. The relative amount of primary antibody was detected with peroxidase-conjugated secondary antibody. Densitometry was used to quantify protein abundance. Similar procedures were carried out with antibodies against PTEN (Sigma Chemical Co), cytochrome *c*, Histone(H3), p65, *β*-actin (all from Santa Cruz Biotechnology), cleaved caspase-3, cleaved caspase-8, cleaved caspase-9, COX IV (all from Cell signaling Technology, Danvers, MA, USA) and Bax.

### Statistical analysis

All experiments were performed at least in triplicate. Results were expressed as mean±S.D. and analyzed by unpaired *t*-test or ANOVA in which multiple comparisons were carried out using the method of least significant difference. The survival data was dissected by log-rank test using SPSS 17.0 software (Chicago, IL, USA). Differences were considered significant if the probability of the difference occurring by chance was <0.05 (*P*<0.05).

## Figures and Tables

**Table 1:** Thirty chosen miRNAs and their sequences

*Name*	*Sequence*
mmu-miR-181b-1	5′-AACAUUCAUUGCUGUCGGUGGGU-3′
mmu-miR-423	5′-AGCUCGGUCUGAGGCCCCUCAGU-3′
mmu-miR-337	5′-UCAGCUCCUAUAUGAUGCCUUU-3′
mmu-miR-370	5′-GCCUGCUGGGGUGGAACCUGGU-3′
mmu-miR-379	5′-UGGUAGACUAUGGAACGUAGG-3′
mmu-miR-376b	5′-AUCAUAGAGGAACAUCCACUU-3′
mmu-miR-682	5′-CUGCAGUCACAGUGAAGUCUG-3′
mmu-miR-412	5′-UUCACCUGGUCCACUAGCCG-3′
mmu-miR-153	5′-UUGCAUAGUCACAAAAGUGAUC-3′
mmu-miR-681	5′-CAGCCUCGCUGGCAGGCAGCU-3′
mmu-miR-27b	5′-UUCACAGUGGCUAAGUUCUGC-3′
mmu-miR-195	5′-UAGCAGCACAGAAAUAUUGGC-3′
mmu-miR-711	5′-GGGACCCGGGGAGAGAUGUAAG-3′
mmu-miR-719	5′-AUCUCGGCUACAGAAAAAUGUU-3′
mmu-miR-16-1	5′-CCAGUAUUGACUGUGCUGCUGA-3′
mmu-miR-15a	5′-UAGCAGCACAUAAUGGUUUGUG-3′
mmu-miR-33	5′-GUGCAUUGUAGUUGCAUUGCA-3′
mmu-miR-615	5′-UCCGAGCCUGGGUCUCCCUCUU-3′
mmu-miR-688	5′-UCGCAGGCGACUACUUAUUC-3′
mmu-miR-690	5′-AAAGGCUAGGCUCACAACCAAA-3′
mmu-miR-691	5′-AUUCCUGAAGAGAGGCAGAAAA-3′
mmu-miR-693	5′-CAGCCACAUCCGAAAGUUUUC-3′
mmu-miR-301	5′-CAGUGCAAUAGUAUUGUCAAAG-3′
mmu-miR-133a-3	5′-UUUGGUCCCCUUCAACCAGCUG-3′
mmu-miR-345	5′-GCUGACCCCUAGUCCAGUGCUU-3′
mmu-miR-145	5′-GUCCAGUUUUCCCAGGAAUCCCU-3′
mmu-miR-143	5′-UGAGAUGAAGCACUGUAGCUC-3′
mmu-miR-146b	5′-UGAGAACUGAAUUCCAUAGGCU-3′
mmu-miR-669b	5′-AGUUUUGUGUGCAUGUGCAUGU-3′
mmu-miR-695	5′-AGAUUGGGCAUAGGUGACUGAA-3′

## Abbreviations

miRNAs: microRNAs
I/R: ischemia–reperfusion
HIF-1: hypoxia-inducible factor-1
PTEN: phosphatase and tensin homolog
NF-*κ*B: nuclear factor kappa B
TUNEL: TdT-mediated dUTP nick end-labeling
TNF-*α*: tumor necrosis factor-*α*
IEC: intestinal epithelial cell
HRE: hypoxia responsive element
HREm: hypoxia responsive element-deletion mutant
ROS: reactive oxygen species
