# Supplementary figures and images for: MicroRNA-682-mediated downregulation of PTEN in intestinal epithelial cells ameliorates intestinal ischemia–reperfusion injury
Source: Cell Death Dis. 2016 Apr 28;7(4):e2210–. doi: 10.1038/cddis.2016.84 (PMC4855663; doi:10.1038/cddis.2016.84)

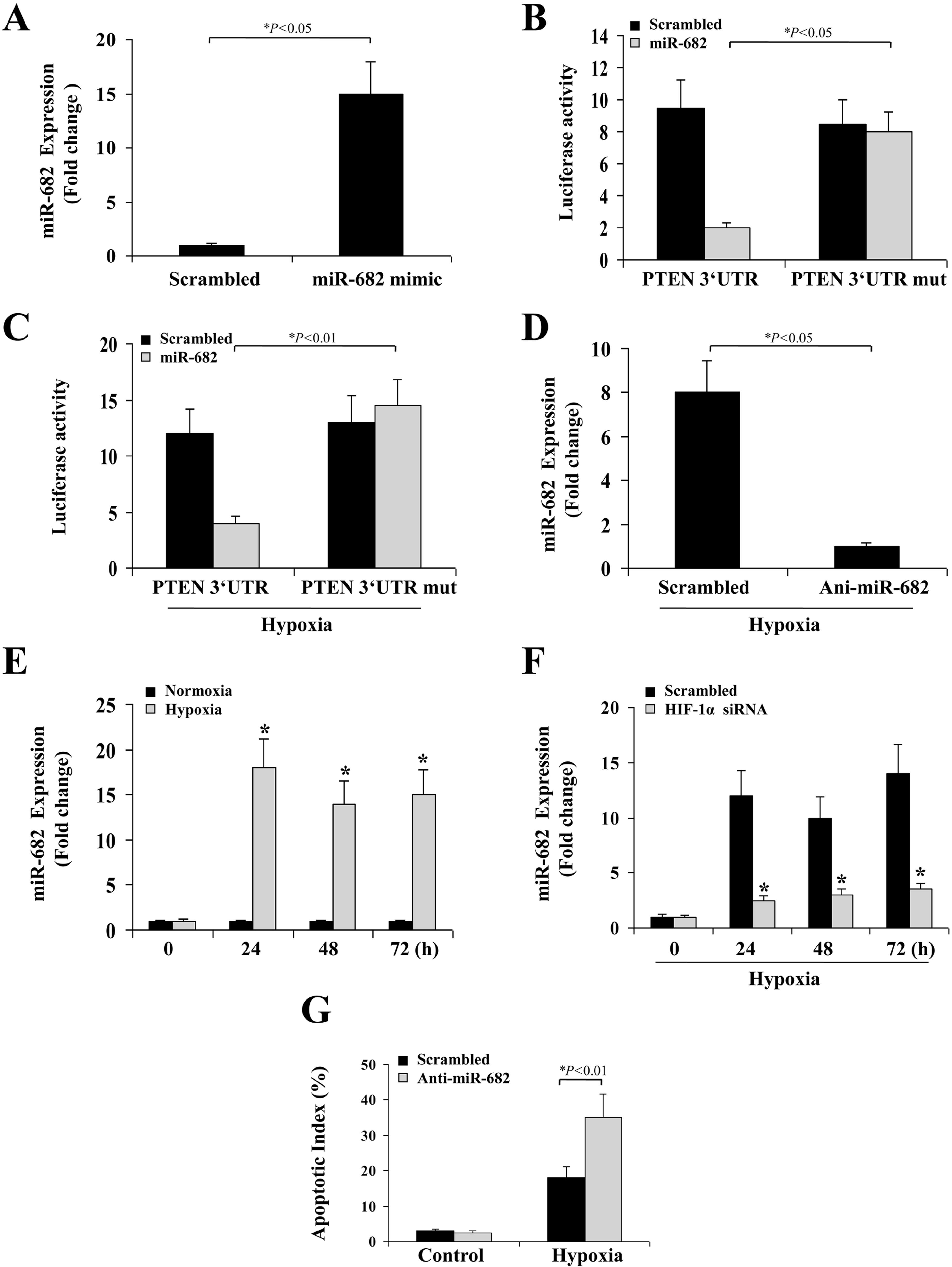

Supplement: Supplementary Figure 1 [file cddis201684x1.tif]

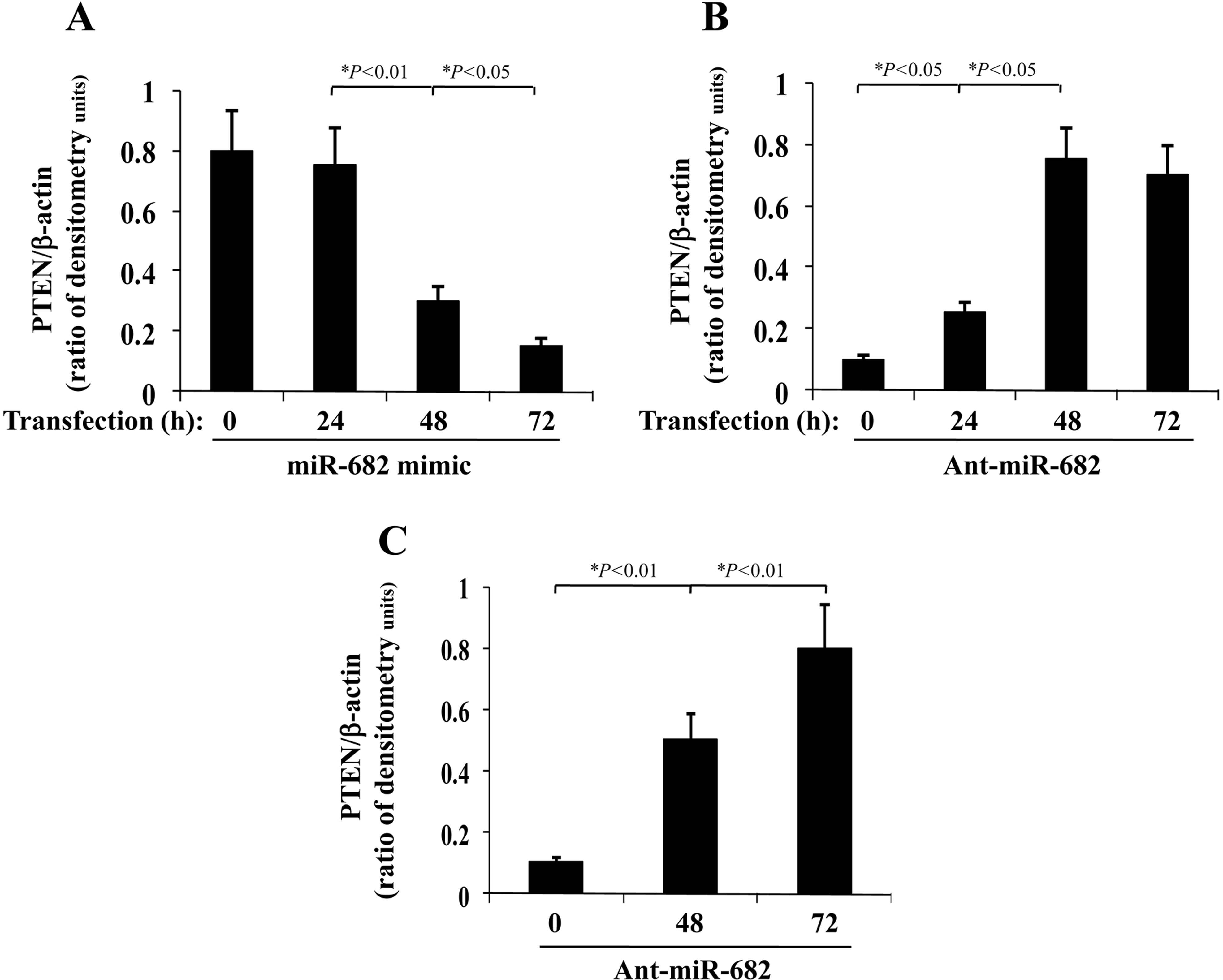

Supplement: Supplementary Figure 2 [file cddis201684x2.tif]

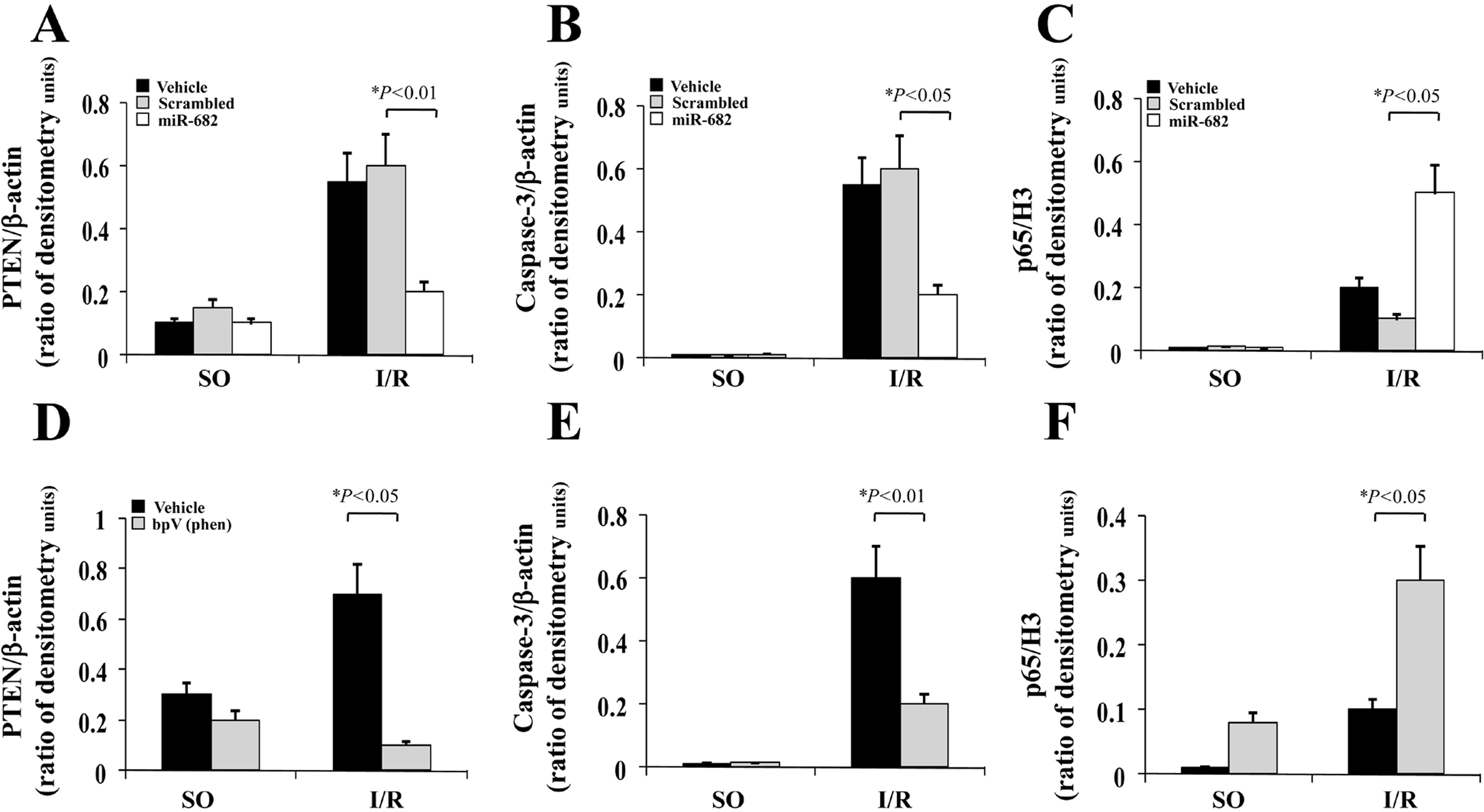

Supplement: Supplementary Figure 3 [file cddis201684x3.tif]

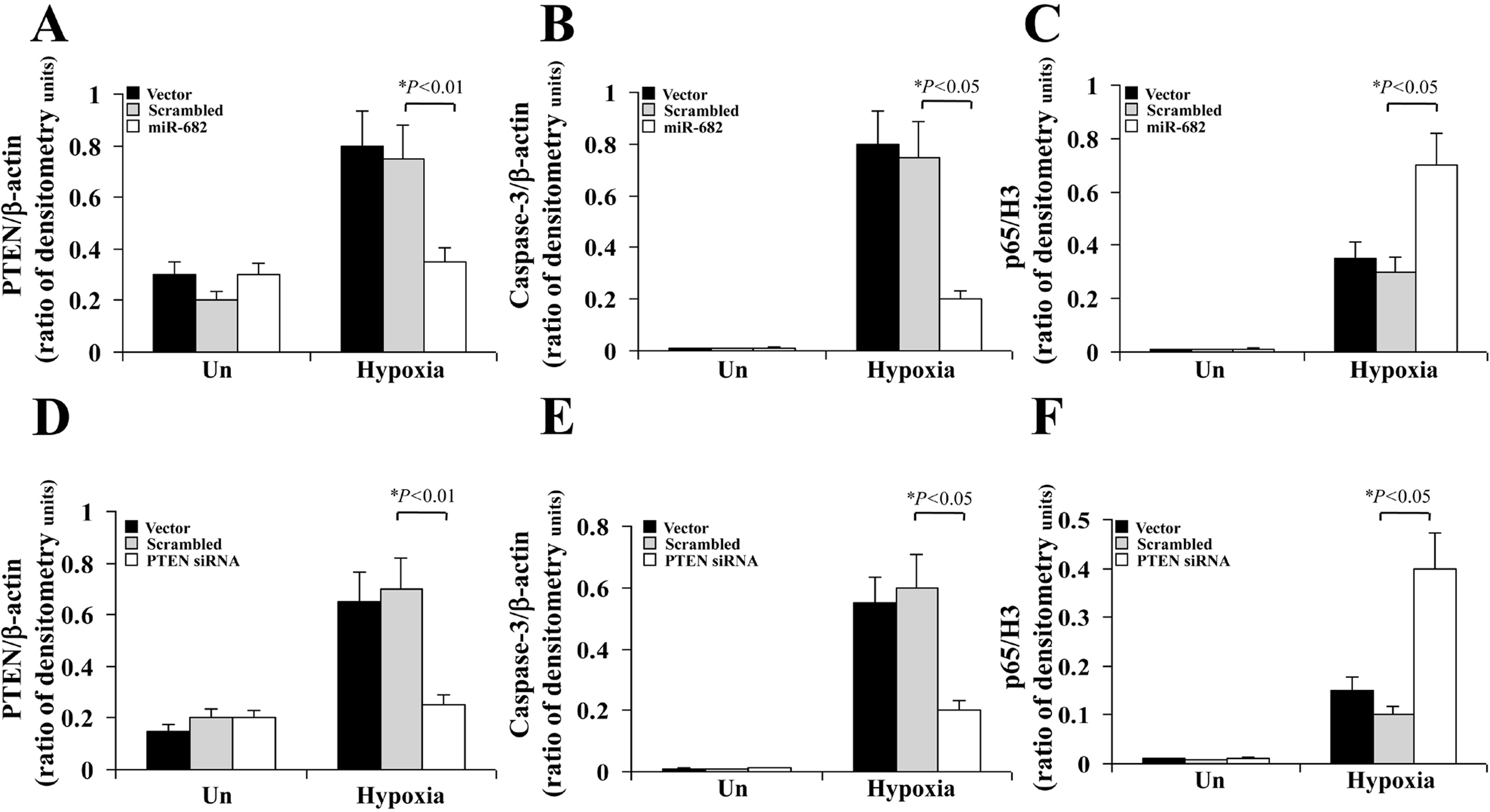

Supplement: Supplementary Figure 4 [file cddis201684x4.tif]

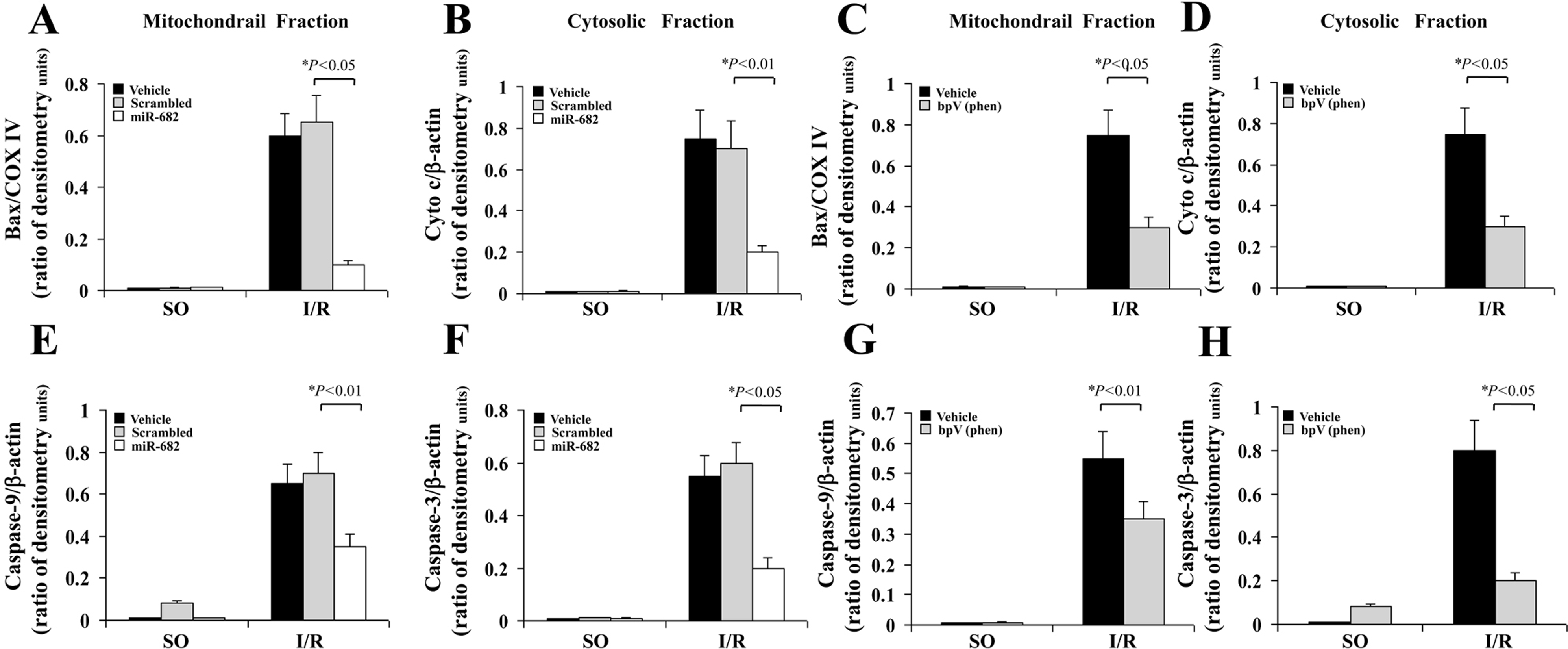

Supplement: Supplementary Figure 5 [file cddis201684x5.tif]

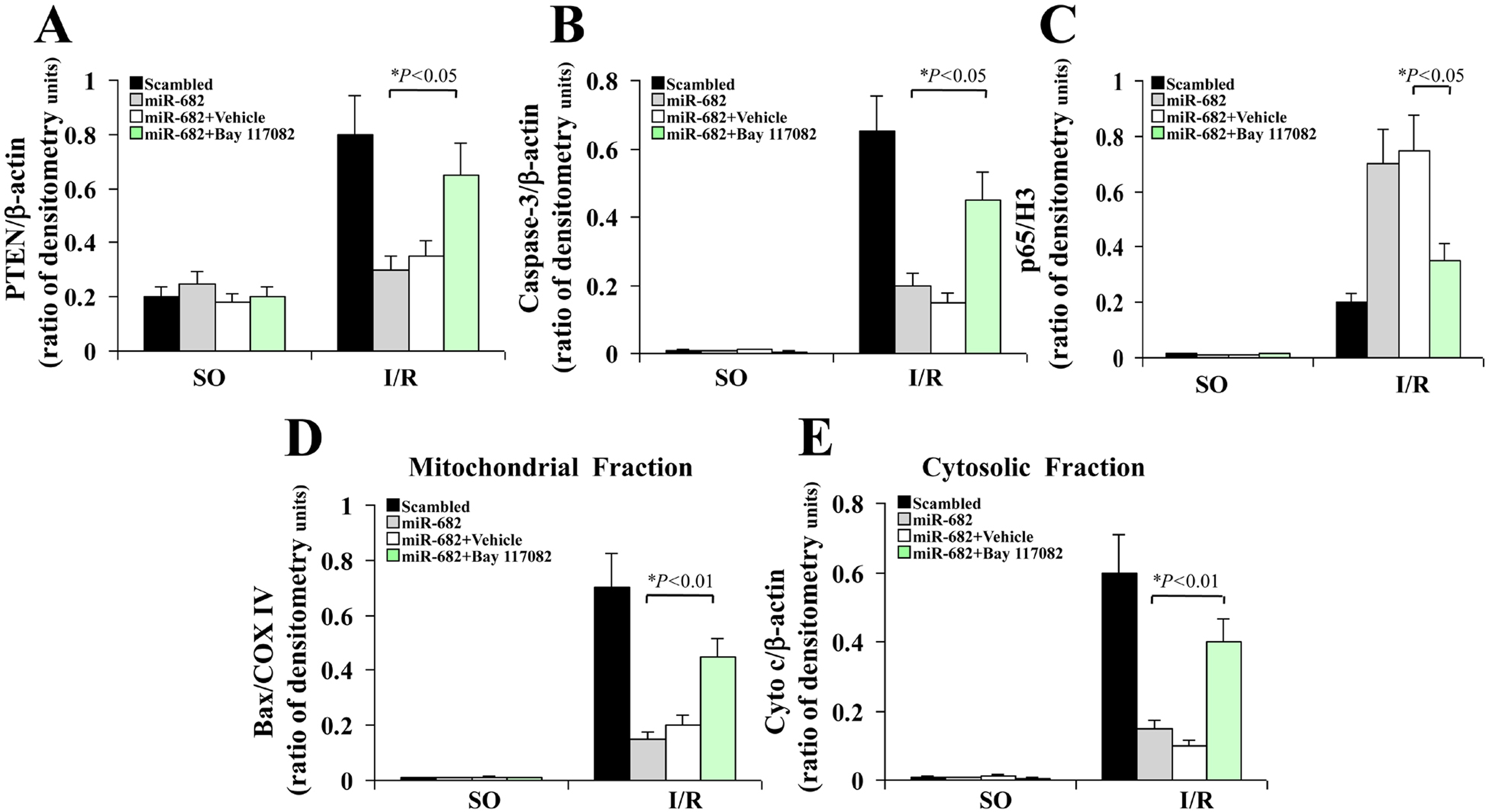

Supplement: Supplementary Figure 6 [file cddis201684x6.tif]
