# Supplementary material for: MicroRNA-682-mediated downregulation of PTEN in intestinal epithelial cells ameliorates intestinal ischemia–reperfusion injury
Source: Cell Death Dis. 2016 Apr 28;7(4):e2210–. doi: 10.1038/cddis.2016.84 (PMC4855663; doi:10.1038/cddis.2016.84)
Supplement: Supplementary Information [file cddis201684x7.doc]

Supplementary Figure 1

(A) Real-time PCR of miR-682 expression. RNA from CCC-HIE-2 cells transfected with or without miR-682 mimic. Values are means±SD. Three independent experiments were performed. (B) Luciferase reporter assay was conducted using constructs with the PTEN 3′UTR or PTEN 3′UTR mutant. CCC-HIE-2 cells were cotransfected with these constructs along with the scrambled or miR-682 mimic. Three independent experiments were performed. (C) Luciferase reporter assay was conducted using constructs with the PTEN 3′UTR or PTEN 3′UTR mutant. CCC-HIE-2 cells were cotransfected with these constructs along with the scrambled or miR-682 mimic for 48 hours under hypoxia. Three independent experiments were performed. (D) Expression of miR-682 inhibited by anti-miR-682 under hypoxia. CCC-HIE-2 cells treated with anti-miR-682, were incubated under hypoxia (1% oxygen) for 48 hours to extract RNA for real-time PCR analysis of miR-682. Fold changes relative to cells with scrambled. (E) Induction of miR-682 in the different time points under hypoxia. CCC-HIE-2 cells were incubated under hypoxia (1% oxygen) for 12-48 hours to extract RNA for real-time PCR analysis of miR-682. Fold changes relative to cells without hypoxia treatment. *P<0.05 (F) Induction of miR-682 in the different time points under hypoxia. CCC-HIE-2 cells treated with HIF-1α siRNA or scrambled were incubated under hypoxia (1% oxygen) for 12-48 hours to extract RNA for real-time PCR analysis of miR-682. Fold changes relative to cells with scrambled. *P<0.05. (G) Apoptotic index was measured by counting a minimum of 20 randomly selected villi and crypts in the sections following TUNEL staining. The index was obtained by dividing the TUNEL positive cells by the total number of cells. *P<0.05 versus SO. #P<0.05 versus Scrambled. Values are means±SD, n=3 in each group.

Supplementary Figure 2

(A)The densitometric ratio of PTEN/β-actin of western blotting from cells with miR-682 mimic in Figure 3B is indicated. The values are expressed as means± SD. Four independent experiments were performed. (B) The densitometric ratio of of PTEN/β-actin of western blotting from cells with anti-miR-682 in Figure 3D is indicated. The values are expressed as means± SD. Four independent experiments were performed. (C) The densitometric ratio of PTEN/β-actin of western blotting from cells with anti-miR-682 in Figure 3F is indicated. The values are expressed as means± SD. Four independent experiments were performed.

Supplementary Figure 3

(A) (B) (C) The densitometric ratio of PTEN/β-actin, Caspase-3/β-actin, or p65/H3 of western blotting from Figure 5F is indicated. The values are expressed as means± SD. Three independent experiments were performed. (D) (E) (F) The densitometric ratio of PTEN/β-actin, Caspase-3/β-actin, or p65/H3 of western blotting from Figure 5H is indicated. The values are expressed as means± SD. Three independent experiments were performed.

Supplementary Figure 4

(A) (B) (C) The densitometric ratio of PTEN/β-actin, Caspase-3/β-actin, or p65/H3 of western blotting from Figure 6E is indicated. The values are expressed as means± SD. Three independent experiments were performed. (D) (E) (F) The densitometric ratio of PTEN/β-actin, Caspase-3/β-actin, or p65/H3 of western blotting from Figure 6F is indicated. The values are expressed as means± SD. Three independent experiments were performed.

Supplementary Figure5

(A) (B) The densitometric ratio of Bax/COX IV, Cyto c/β-actin of western blotting from Figure 7A is indicated. The values are expressed as means± SD. Three independent experiments were performed. (C) (D) The densitometric ratio of Bax/COX IV, Cyto c/β-actin of western blotting from Figure 7C is indicated. The values are expressed as means± SD. Three independent experiments were performed. (E)(F) The densitometric ratio of Caspase-9/β-actin, Caspase-3/β-actin of western blotting from Figure 7B is indicated. The values are expressed as means± SD. Three independent experiments were performed. (G)(H) The densitometric ratio of Caspase-9/β-actin, Caspase-3/β-actin of western blotting from Figure 7D is indicated. The values are expressed as means± SD. Three independent experiments were performed.

Supplementary Figure6

(A) (B) (C) The densitometric ratio of of PTEN/β-actin, Caspase-3/β-actin, or p65/H3 of western blotting from Figure 8E is indicated. The values are expressed as means± SD. Three independent experiments were performed. (D) (E) The densitometric ratio of Bax/COX IV, Cyto c/β-actin of western blotting from Figure 8F is indicated. The values are expressed as means± SD. Three independent experiments were performed.
